# To: Ventriculitis incidence and outcomes in patients with aneurysmal subarachnoid hemorrhage: a prospective observational study

**DOI:** 10.62675/2965-2774.20250088

**Published:** 2025-10-31

**Authors:** Julya Santana Alves de Barroso, Henri Dourado de Almeida, Amanda Cirilo de Oliveira Lins, Jorge Fernando Pereira Silva, Achilles de Souza Andrade, Johnnatas Mikael Lopes

**Affiliations:** 1 Universidade Federal do Vale São Francisco Paulo Afonso BA Brazil Universidade Federal do Vale São Francisco - Paulo Afonso (BA), Brazil.; 2 Universidade Federal da Paraíba Department of Medicine João Pessoa PB Brazil Department of Medicine, Universidade Federal da Paraíba - João Pessoa (PB), Brazil.

## To the Editor

Aneurysmal subarachnoid hemorrhage (ASH) is a worldwide health burden with high fatality and permanent disability rates. Aneurysmal subarachnoid hemorrhage accounts for only 5% of all strokes, but it has high mortality and permanent disability rates.^(1)^ Ventriculostomy, as a treatment method for ASH, may produce complications like inflammation of the ependymal lining of the cerebral ventricles, called ventriculitis, due to infection of the temporary external ventricular drain catheter.^(1)^

Ventriculostomy treatment may produce complications like inflammation of the ependymal lining of the cerebral ventricles, called ventriculitis, due to infection of the temporary external ventricular drain (EVD) catheter.^(1)^ The study by Turon et al.,^(2)^ in Critical Care Science, addresses a topic of great relevance to critical care and presents interesting results. However, analytical elements limit the findings of risk association for post-discharge outcomes beyond what Veiga et al.^(3)^ highlighted in their editorial. However, it is possible to answer Veiga et al.'s^(3)^ first question about the power of the sample to detect differences. To do this, we will assume that the study should be analyzed using Cox regression, and we will explain why later. Thus, assuming that each group is equal in size, with a significance level of 5% and a test power of 80%, it would be necessary to observe 3,457 outcomes for a minimum difference of 10% in the hazard ratio (HR) and 457 outcomes for a minimum difference of 30% in the HR between the groups. It should be noted that these values do not constitute a sample size, which should be much larger than the number of outcomes.^(4)^

Furthermore, the inferential limitation focuses on two important methodological points. The first point is the loss to follow-up over 12 months, which makes it impossible to use the odds ratio (OR) estimate.^(5)^ Both groups had more than 20% sample losses at follow-up. This tells us that the sample at the end of the study changed and, therefore, so did the risk estimate during the period.^(5)^ The second methodological aspect is that using OR to estimate the effect in prospective studies produces oversized interval measures, which can interfere with the significant risk effect, as can be seen for the variables age, endovascular coiling, and infection associated with ventriculostomy. The solution to this problem is to analyze the data as a survival study using Cox regression and use HR as an effect measure. We suggest that prospective studies with a loss to follow-up of more than 10%, which occurs in long follow-up studies, use Cox regression to estimate the effect on the outcome. In addition, it is important to observe whether the factors analyzed change their action during follow-up, which is called (dis)proportional risk. These aspects limit the usefulness of the model presented, especially in future systematic reviews with meta-analysis and in decision-making in health.

## References

[1] D’Souza S (2015). Aneurysmal subarachnoid hemorrhage. J Neurosurg Anesthesiol.

[2] Turon R, Kurtz P, Rynkowski C, Petterson L, Gonçalves B, Caro V (2025). Ventriculitis incidence and outcomes in patients with aneurysmal subarachnoid hemorrhage: a prospective observational study. Crit Care Sci.

[3] Veiga VC, Kalil A, Soares PH, Póvoa P (2025). Ventriculostomy-associated infections: a healthcare issue in the neurointensive care unit. Crit Care Sci.

[4] Schoenfeld DA (1983). Sample-size formula for the proportional-hazards regression model. Biometrics.

[5] Knol MJ, Le Cessie S, Algra A, Vandenbroucke JP, Groenwold RH (2012). Overestimation of risk ratios by odds ratios in trials and cohort studies: alternatives to logistic regression. CMAJ.

